# Chronic conditions in women: the development of a National Institutes of health framework

**DOI:** 10.1186/s12905-023-02319-x

**Published:** 2023-04-06

**Authors:** Sarah M. Temkin, Elizabeth Barr, Holly Moore, Juliane P. Caviston, Judith G. Regensteiner, Janine A. Clayton

**Affiliations:** 1 Office of Research On Women’s Health, National Institutes of Health, Bethesda, MD USA; 2 National Institute On Drug Abuse National Institutes of Health, Bethesda, MD USA; 3 Department of Medicine, Ludeman Family Center for Women’s Health Research, University of Colorado Anschutz School of Medicine, Aurora, CO USA

**Keywords:** Women’s health, Chronic disease, Multimorbity, Sex differences, Gender

## Abstract

Rising rates of chronic conditions were cited as one of the key public health concerns in the Fiscal Year (FY) 2021 U.S. Senate and House of Representatives appropriations bills, where a review of current National Institutes of Health (NIH) portfolios relevant to research on women’s health was requested. Chronic conditions were last defined by the US Department of Health and Human Services (HHS) in 2010. However, existing definitions of chronic conditions do not incorporate sex or gender considerations. Sex and gender influence health, yet significant knowledge gaps exist in the evidence-base for prevention, diagnosis, and treatment of chronic diseases amongst women. The presentation, prevalence, and long-term effects of chronic conditions and multimorbidity differs in women from men. A clinical framework was developed to adequately assess the NIH investment in research related to chronic conditions in women. The public health needs and NIH investment related to conditions included in the framework were measured. By available measures, research within the NIH has not mapped to the burden of chronic conditions among women. Clinical research questions and endpoints centered around women can be developed and implemented; clinical trials networks with expanded or extended eligibility criteria can be created; and data science could be used to extrapolate the effects of overlapping or multiple morbidities on the health of women. Aligning NIH research priorities to address the specific needs of women with chronic diseases is critical to addressing women’s health needs from a life course perspective.

## Background

Rising rates of “chronic debilitating conditions among women” were one of three key public health concerns cited in the Fiscal Year (FY) 2021 U.S. Senate and House of Representatives appropriations bills, where a review of current National Institutes of Health (NIH) portfolios and an expert convening related to women’s health research was requested. The NIH’s Office of Research on Women’s Health (ORWH) responded to this congressional request by reviewing the current NIH activities on the priority topics, as well as collecting input on the priority areas from experts in women’s health and members of the public. Members of the NIH Advisory Committee on Research on Women’s Health (ACRWH) formed a working group to review NIH activities, plan an interdisciplinary conference, and identify opportunities for future NIH-supported women’s health research. On October 20, 2021, ORWH Advancing NIH Research on the Health of Women: A 2021 Conference (https://orwh.od.nih.gov/research/2021-womens-health-research-conference) was co-hosted by ORWH and the ACRWH. This manuscript provides an overview of the response to Congress on the topic of chronic conditions in women, including an overview of the public health needs, current research, and opportunities for future research to advance our knowledge on this topic.

### Main text

#### Defining chronic conditions

Accurate definitions of chronic conditions allow accurate public health surveillance and population monitoring. However, existing definitions of chronic conditions vary widely with heterogeneity in characteristics such as the duration or latency, need for medical attention, effect on function, pathology, departure from well-being, noncontagious nature, multiple risk factors, and amenability to cure [1]. Chronic conditions were last defined by the U.S. Department of Health and Human Services (HHS) in 2010 as “conditions that last a year or more and require ongoing medical attention and/or limit activities of daily living” [2]. The Centers for Medicare and Medicaid Services (CMS) defines chronic conditions (Table 1) within its beneficiaries as receiving a service or treatment for one of 21 specific conditions [3]. The World Health Organization defines chronic conditions as”health problems that require ongoing management over a period of years or decades” [4].

**Table 1:** CMS-defined chronic conditions

Alcohol Abuse	Drug Abuse/Substance Abuse
Alzheimer’s Disease and Related Dementia	Heart Failure
Arthritis (Osteoarthritis and Rheumatoid)^a^	Hepatitis (Chronic Viral B & C)
Asthma^a^	HIV/AIDS
Atrial Fibrillation	Hyperlipidemia (High cholesterol)
Autism Spectrum Disorders	Hypertension (High blood pressure)^a^
Cancer (Breast, Colorectal, Lung, and Prostate)	Ischemic Heart Disease
Chronic Kidney Disease	Osteoporosis^a^
Chronic Obstructive Pulmonary Disease	Schizophrenia and Other Psychotic Disorders
Depression^a^	Stroke
Diabetes	

From: https://www.cms.gov/Research-Statistics-Data-and-Systems/Statistics-Trends-and-Reports/Chronic-Conditions/CC_Main

^a^Higher prevalence in women

#### Multiple chronic conditions

Comorbidity was originally defined as”any distinct additional entity that has existed or may occur during the clinical course of a patient who has the index disease under study,” [5] while multimorbidity is defined as the simultaneous occurrence of two or more diseases that may or may not share a causal link [6]. Comorbidity is the preferred term when there is a specified index condition, while multimorbidity is preferred where an individual has any two or more conditions [7]. Data from the 2018 National Health Interview Survey (NHIS) estimate more than half (51.8%) of adults had at least one of 10 commonly diagnosed chronic conditions (arthritis, cancer, chronic obstructive pulmonary disease, coronary heart disease, asthma, diabetes, hepatitis, hypertension, stroke, and renal dysfunction), and 27.2% of U.S. adults had multiple chronic conditions [8]. A cross-sectional analysis of the National Health and Nutrition Examination Survey (NHANES) demonstrated 59.6% of U.S. civilians 20 years or older had multimorbidity with two or more chronic conditions, 38.5% had three or more chronic conditions, and 22.7% had more than four chronic conditions [9].

### Disability and chronic conditions in women

With the aging of the U.S. population and longer life expectancies of women compared to men, chronic conditions pose an increasingly significant burden on the health of women [10]. Although sex and gender differences in the prevalence of chronic conditions have been documented, existing definitions of chronic conditions do not incorporate sex or gender considerations. Sex-disaggregated CMS data for the twenty-one CMS-defined chronic conditions demonstrate six conditions that occur more frequently in women: hypertension, arthritis, depression, dementia, asthma, and osteoporosis [11]. These data include fee-for-service beneficiaries, excluding Medicare Advantage enrollees. The prevalence of multi-morbidity is higher among female participants within NHANES [9].

### The influence of sex and gender

Understanding the influences of sex and gender on health is critical so that diagnoses and treatments are made accurately for women and men. Sex is a multidimensional construct based on a cluster of anatomical and physiological traits that include external genitalia, secondary sex characteristics, gonads, chromosomes, and hormones [12]. Gender is a multidimensional construct that can encompass gender identity, gender expression, gender roles and norms, gender power relations, and gender inequities [12].

The construct of sex as a biological variable refers to the biological differences that influence health. Many sex differences in disease prevalence, manifestation, and response to treatment have been described. The differential effect of androgens, estrogen, and progesterone on vasculature influences the incidence and response treatment for women with heart disease [13, 14]. Sex differences in the innate and adaptive immune system likely influence the risk for certain diseases (e.g., asthma, autoimmunity), as well as response to vaccination and certain cancer therapies [15]. A historic overreliance on male clinical research subjects has, however, left significant gaps in the evidence-base for female-specific prevention, diagnostic, and treatment interventions for chronic diseases [16]. The broad assumption that women’s health is inexorably linked to reproductive health has limited research on sex-specific conditions beyond those linked to reproduction. An evidence-base for sex differences in other conditions (e.g., cardiovascular, metabolic, neurologic diseases) that affect women has therefore been slow to develop. Ample evidence supports the assertion that critical sex differences affect the disease course of many conditions. Chronic disease risk in women accumulates with age and accelerates following menopause corresponding to a decline in reproductive hormone production [17, 18]. However, the effects of hormonal transitions on the natural history of chronic conditions have not been well-described. Few sex-specific guidelines for clinical treatment of chronic conditions exist [19].

The construct of gender and its effects on social, psychological, and cultural roles influence the development, diagnosis, and treatment of chronic diseases and multimorbidity in women [9]. Lower socioeconomic status and lower educational attainment are among the many gendered risk factors for chronic disease and multimorbidity that disadvantage women [9]. Gender differences have been documented for patient-provider interactions, such that women’s symptoms are often dismissed, and resultant diagnostic delays for diseases such as cancer and cardiovascular disease (CVD) have been demonstrated [20, 21]. Differences in the clinical presentation of diseases for patients who are women have been documented with symptoms more commonly displayed by women often labeled as “atypical.” For example, during a heart attack, women may experience back pain, dizziness or nausea, while men more often present with chest pain and diaphoresis [22]. Similarly, women can experience nonspecific symptoms (e.g., confusion and general weakness, as opposed to weakness on one side of the body) during a stroke [23]. Even when a diagnosis is made, women may face delays in referral for care or even not be offered care at the same rate as men. For example, late referral for osteoarthritis (based on patient or healthcare professional factors) for women results in worse function at the time of joint replacement surgery, impacting the level of function that women achieve after surgery [24]. Stigma around menstrual disorders and other high-burden diseases of the female genital track additionally contributes to societal tolerance of inadequate treatments and limited research investment [25].

Although both sex and gender affect health profoundly, these variables are not routinely disaggregated in reporting of clinical research. A recent evaluation estimated that fewer than a third of published studies reported at least one outcome by sex or explicitly included sex as a covariate in statistical analysis; with explanations for the exclusion of sex in analyses rare [26]. Disentangling the influence of either sex, gender or both on disease is a complex undertaking that has been limited to date. Evolving social norms have shifted definitions and few tools to measure sex and gender have been available and validated [12]. Cancers that affect both sexes not only are influenced by smoking and alcohol consumption, but also may have hormonal drivers that affect incidence [27]. Women are still more likely than men to have their symptoms of CVD ascribed to psychiatric causes, although sex differences in cardiovascular structure and function, as well as hormonal influences, may be associated with worse CVD outcomes for women [28].

Additional social factors magnify the burden of chronic diseases for women with intersectional identities, such identity with an underrepresented racial and ethnic group or socioeconomic disadvantage [29]. Importantly, some female-specific chronic conditions are more prevalent in women in certain historically underrepresented racial and ethnic groups (e.g., higher incidence of uterine fibroids in Black women) [30].

### Multimorbidity in women

**Fig. 1:**
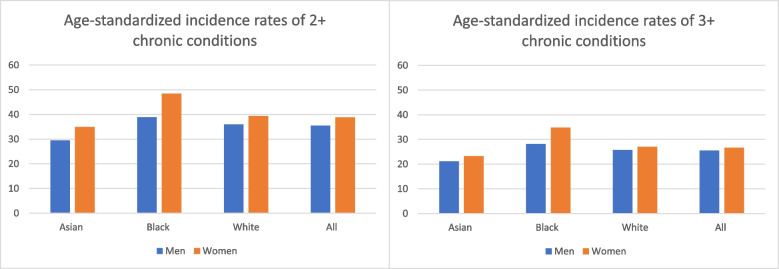
Age- standardized incidence rates of the accumulation of morbidity and multimorbidity disaggregated by race and gender. Adapted from St Sauver JL, et al. BMJ Open 2015;5:e006413 http://dx.doi.org/10.1136/bmjopen-2014-006413

In addition to a higher prevalence of multimorbidity in women, the pattern of accumulating morbidity, meaning which initial chronic conditions are diagnosed and how conditions are additive, differs by sex and gender [31]. The “networks” of morbidity in women more frequently cross multiple organ systems [32]. In women with multimorbidity, the interactions among conditions are poorly understood and often inadequately treated [9]. Differences among racial and ethnic populations, as well as the effects of other intersecting social determinants of health in the prevalence of multimorbidity, remain less explored and controversial [6]. In addition to the influence of sex and gender, race and ethnicity are associated with the incidence of multimorbidity (Fig. 1), with Black women having the highest burden of disease [33].

### Life course perspective

A life course perspective (particularly information on pregnancy and menopause) is useful when considering women’s health care. Hypertensive disorders during pregnancy, for example, can increase the risk of developing hypertension within three years after giving birth [34]. Menstrual abnormities with or without a diagnosis of polycystic ovarian syndrome (PCOS) prior to menopause is associated with higher risk of CVD later in life [35, 36]. Although the Women's Health Initiative (WHI), which was the largest randomized, placebo‐controlled trial evaluating menopausal hormone therapy (MHT) in postmenopausal women, demonstrated increased risk of adverse events with MHT, subsequent secondary analyses have demonstrated that these risks differ by hormonal preparation, age, and time since menopause [37, 38]. The ideal dosing, delivery method, formulation, and timing of hormonal therapy to mitigate CVD risk remains unknown, as is the risk to benefit ratio of treating menopausal symptoms [39].

### Development of a framework for chronic conditions in women

Due to the lack of definitions of chronic conditions specific to women, a framework was created for the purpose of fulfilling the congressional request for NIH portfolio analysis on this topic. This framework (Table 2) categorized chronic debilitating conditions in women into the following: (1) female-specific, (2) more common in women and/or morbidity is greater for women, (3) potentially understudied in women, and (4) high morbidity for women. Disability-adjusted life years (DALYs) defined by the WHO as “the loss of the equivalent of one year of full health,” were used as a metric by which to measure the burden of disease [40]. DALYs for a disease or health condition are the sum of the years of life lost due to premature mortality, and the years lived with a disability due to prevalent cases of the disease or health condition in a population. Although DALYs are unable to quantify limitations of functioning, activity, or social participation, they provide an available metric by which to compare the health impact in a standardized fashion across multiple medical conditions [41]. DALYs were obtained from the Institute for Health Metrics and Evaluation (IHME), Global Burdens of Disease (https://ghdx.healthdata.org/) and wherever possible for diseases and health conditions that overlap with NIH’s Research, Condition, and Disease Categorization (RCDC) system – the official system, of record for annual NIH funding on specific research topics and conditions.

**Table 2:**
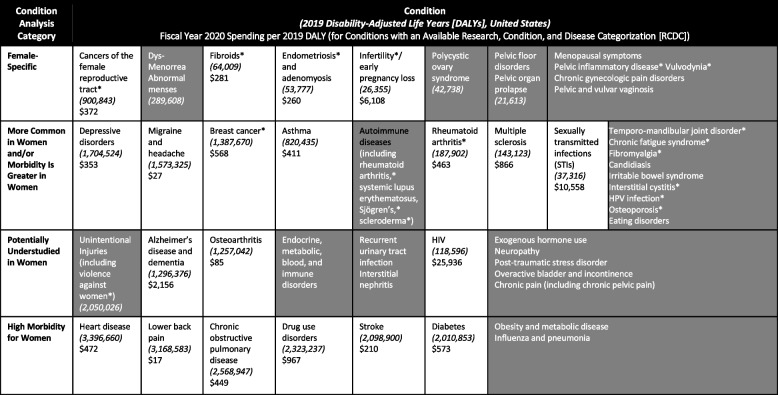
A framework for the consideration of chronic debilitating conditions in women

^a^Per Manual Categorization System-Women’s Health reporting guidance, the starred RCDCs are considered particularly relevant to women’s health

Gray boxes represent conditions without an RCDC category (funding estimates not available)

Sources: DALYs obtained from http://ghdx.healthdata.org/gbd-results-tool; RCDC spending obtained from https://report.nih.gov/funding/categorical-spending#/

Specific challenges persist related to the diagnosis and treatment of female-specific chronic conditions, which are not always included in current multimorbidity indices. Many female-specific conditions remain underdiagnosed with an insufficient evidence-base for diagnosis and treatment, which presumably negatively affects outcomes [42]. Receipt of a definitive diagnosis of endometriosis typically requires 4 to 11 years from symptom onset [43]. PCOS increases the risk for other chronic health problems (e.g., depression, anxiety, and eating disorders), can have multiple phenotypes, and may be linked with obesity, infertility, and endometrial cancer and other malignancies [44, 45]. Studies to date of medical treatment for fibroids, the most common gynecological disorder, have been of low or moderate quality [46].

Many common chronic conditions are not female-specific but occur at substantially higher rates in women compared to men. Women constitute nearly 80% of the population affected by autoimmune disease and bear a disproportionately high morbidity associated with this spectrum of conditions [47]. Other disorders, such as depression, are thought to be disproportionately high among women for a combination of innate factors (e.g., fluctuations in hormones), as well as social factors (e.g., high rates of exposure to intimate partner violence) [48, 49].

Other chronic conditions are not female-specific and do not have sex-specific etiologies but remain studied more commonly in men than in women. These include disorders such as HIV and post-traumatic stress disorder that affect women yet remain socioculturally associated with men and largely understudied in women [50]. Other conditions such as urinary incontinence are common (nearly half of older women experience incontinence) and significantly impact quality of life, yet are infrequently included in women’s health research agendas [51].

Many chronic conditions with high morbidity in women go unrecognized as significant women’s health issues. CVD is the most common cause of death among women; and yet, among American women and health care providers, only 45% of women knew that CVD is the leading cause of death among women [52]. The prevalence of chronic obstructive pulmonary disease (COPD) among women has equaled that of men since 2008, due in part to increased tobacco use among women. Despite this, women remain under-diagnosed compared to men and receive fewer spirometry tests and medical consultations [53]. Among patients with diabetes, there is a higher prevalence of obesity and poorer blood pressure control in women than men, both of which can cause cardiovascular complications. Diabetes is also a stronger risk factor for stroke in women compared to men [54, 55].

### Ongoing NIH research

The purpose of the NIH Revitalization Act of 1993, which led to the formation of the NIH ORWH was to improve representation of women in clinical research. Today, in overall enrollment to NIH-supported clinical trials, roughly half of participants are women [26]. Yet underrepresentation of female enrollment in clinical trials, when compared with population prevalence, persists in certain conditions that cause substantial morbidity in women, including HIV/AIDS, kidney diseases, and cardiovascular diseases [56]. Disparities in funding also have been described for diseases that affect women. For many diseases that affect primarily one sex, the funding pattern favors those that primarily affect males with respect to burden of the disease within the population. For conditions and disorders that are female-dominant, NIH funding is comparably lower than those conditions that predominantly occur in men. The disparity between actual funding and the disease burden by sex is nearly twice as large for diseases more prevalent in females versus those more prevalent in males [57].

NIH spent 10.8 percent of its funding ($4,466 million) on women’s health research in FY 2020. NIH supports a wide range of research on chronic diseases—covering screening and prevention, diagnostics, treatment and therapeutics, health disparities, and other activities (e.g., mechanisms and pathogenesis). However, no single NIH RCDC code captures the breadth of chronic conditions. The lack of a single RCDC code limited the portfolio analysis in response to the congressionally directed review of NIH research activities. In lieu of a quantitative portfolio review, ratios of FY 2020 NIH spending per DALY was calculated within the framework for chronic debilitating conditions (Table 2) with available RCDC codes. The ratio of FY 2020 NIH spending to 2019 disability-adjusted life years for U.S. women varied widely from $17 per disability-adjusted life years for lower back pain to $25,936 per disability-adjusted life years for. When calculated in this manner, NIH spending was not aligned with the burden of diseases among women.

#### Female-specific conditions

Research on female-specific conditions and diseases remains limited [57]. Scientific inquiry on subjects with such conditions as menopause, endometriosis, or fibroids does not clearly fall under the purview of a single Institute, Center or Office (ICO), and NIH receives fewer unsolicited investigator-initiated grant applications addressing these female-specific conditions compared with other NIH-supported research topics. Without this foundational knowledge, gaps remain in providing high-quality, evidence-based care for women.

Efforts are underway to advance knowledge about female-specific disorders in some areas of health. The Gynecologic Health and Disease Branch was established within the *Eunice Kennedy Shriver* National Institute of Child Health and Human Development (NICHD) in 2012 to support studies of gynecologic disorders, including endometriosis, adenomyosis, fibroids, and polycystic ovary syndrome [58]. In 2021, the National Heart, Lung, and Blood Institute (NHLBI), ORWH, and other NIH partners hosted the Cardiovascular Risk Across the Lifespan for Polycystic Ovary Syndrome Workshop (https://www.nhlbi.nih.gov/events/2021/cardiovascular-risk-across-lifespan-polycystic-ovary-syndrome-workshop), a two-day virtual workshop to review the state of science on CVD across the life course of women with PCOS and identified knowledge gaps and opportunities in PCOS-related CVD research.

### Sex differences in drug metabolism, pathogenesis, and disease manifestations

In 2016, the NIH implemented the Sex as a Biological Variable (SABV) policy, that outlined an expectation “that sex as a biological variable will be factored into research designs, analyses, and reporting in vertebrate animal and human studies” unless a strong justification for a single-sex study exists [59]. ORWH, with support from the National Institute of General Medical Sciences (NIGMS) and Office of the Director (OD), has developed a portfolio of interprofessional education materials to enhance understanding of the SABV policy. The Specialized Centers of Research Excellence (SCORE) on Sex Differences program is a signature program of ORWH that serves, at multiple levels of analysis, to support research to identify the role of biological sex differences on the health of women.

### Multimorbidity

**Fig. 2:**
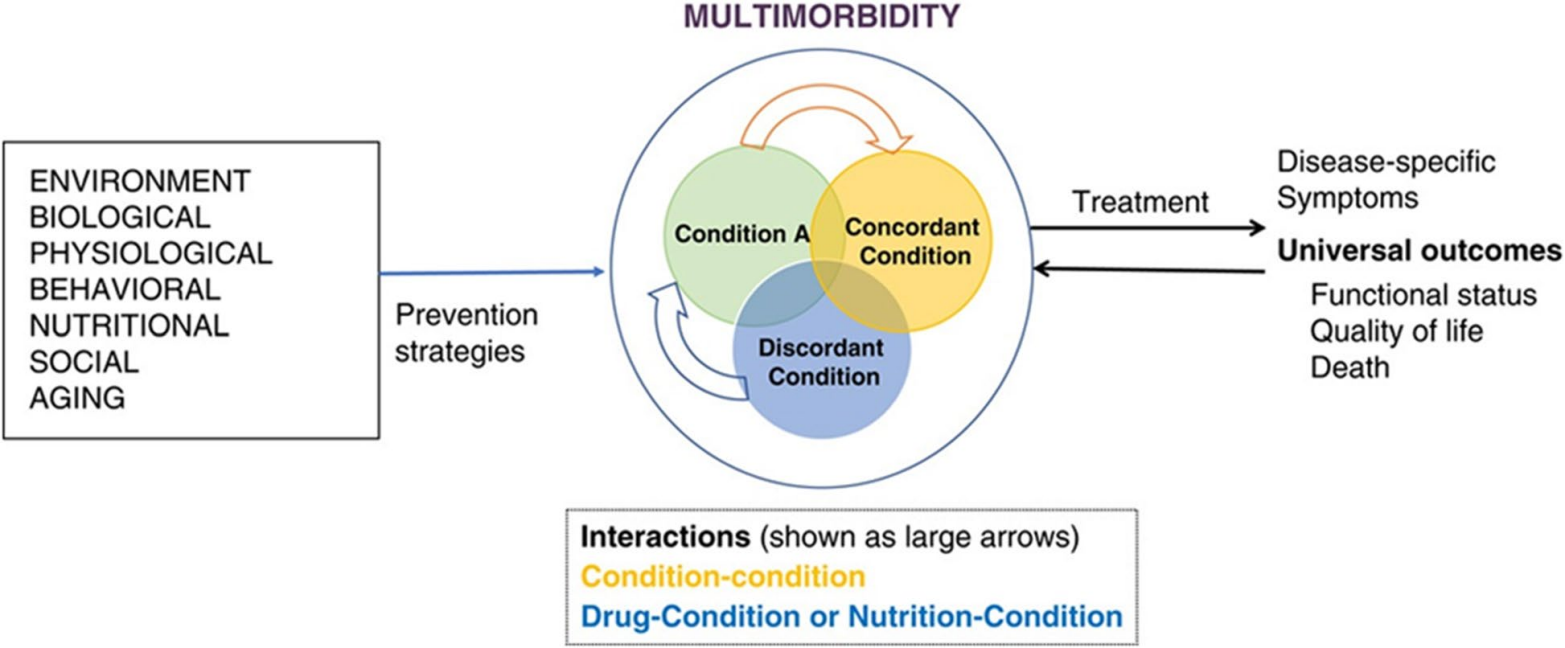
Conceptual model and research framework for multimorbidity, depicting relationships among causal factors, disease conditions and interactions, and outcomes of multimorbidity. Source: Salive ME, Suls J, Farhat T, Klabunde CN. 2021. National Institutes of Health Advancing Multimorbidity Research. Med Care. 2021;59(7):622–624. 10.1097/MLR.0000000000001565. PMID: 33,900,269

In 2018, several NIH ICOs—the National Cancer Institute (NCI), National Institute of Aging (NIA), National Institute on Minority Health and Health Disparities (NIMHD), the Office of Behavioral and Social Sciences Research (OBSSR), and the Office of Disease Prevention (ODP)—held an expert panel workshop entitled, “Measuring Multimorbidity: Matching the Instrument and the Purpose.” From this meeting, a model and research framework for multimorbidity—depicting relationships among causal factors, disease conditions and interactions, and outcomes of multimorbidity—was developed (Fig. 2). This model does not consider sex and gender, yet it serves as a useful tool for conceptualizing multimorbidity [60]. NIA supports several research projects on multimorbidity; including several projects that overlap with women’s health.

### Opportunities for future research on chronic conditions in women

To produce interventions that meet the needs of all women with chronic conditions, sex and gender must be considered in the design, analysis, and reporting of studies. The framework for chronic debilitating conditions in women presented here can be used as a starting point for conceptualizing the diversity of chronic conditions in women and developing standardized reporting and analysis of chronic conditions in women.

### Rethinking preclinical research

Preclinical research and drug development studies have historically predominantly used male animal models and cells [13]. The SABV policy has led to preclinical and discovery knowledge of relevant diseases that affect women. Using SABV as a guiding principle in preclinical research and throughout the research continuum can help address the critical needs in research on the health of women by (1) identifying animal models and ex vivo human models (e.g., explants, organoids) that better reflect human diseases, (2) developing diagnostic tests and criteria that are sex-specific, and (3) understanding the increased risk of adverse events and reduced treatment effectiveness in women. Continued attention to and enforcement of the SABV policy will lead to further understanding of how sex influences physiology and pathophysiology, allowing improvements in disease prevention and treatment strategies in the multitude of conditions that present differently in women and require different treatment in women and men. An in-depth knowledge of the cellular and molecular processes underlying pathophysiology would enhance our understanding of female-specific chronic conditions and may be broadly applicable to studying sex as a biological factor in human health.

### Using data science

The current research paradigm focuses most commonly on a single disease, disorder, or condition with capture of comorbidities [61]. An increased research focus on data science, would enable the exploration of potentially causal associations among multiple coexisting conditions both enhancing our understanding of the accumulation of morbidity and multimorbidity. Common patterns of genetic, biologic, social and/or environmental factors, including sex and gender, and susceptibility to clusters of co-occurring diseases can be identified. Innovative approaches for data science to research on morbidity and multimorbidity in women could include targeted support modeled on other successful NIH initiatives, such as NCI’s Surveillance, Epidemiology, and End Results (SEER database) or NCATS National COVID Cohort Collaborative (N3C) [62, 63]. Advances in automated extraction of participant data can provide an effective alternative to manual disaggregation of data [56]. However, biases in machine learning and artificial intelligence algorithms must be continually critically examined to ensure that societal biases and health inequities are not exacerbated by digital technology [64].

### Redesigning clinical trials

Since the establishment of ORWH, NIH has made significant advances in research focused on women—today approximately half of enrollees in NIH-supported clinical research are women. Although women are now routinely included in clinical research, research is infrequently centered around the health needs and circumstances of women. These specific needs and circumstances include individual-level biological factors such as menstruation, pregnancy, and menopause, as well as social determinants of health such as structural sexism, gendered power relations, and gender differences in health care access and quality [65, 66]. Because of the higher rates of chronic conditions and disability among women, endpoints such as functioning, and quality of life are of critical importance. A number of highly prevalent female-specific chronic conditions such as dysmenorrhea, fibroids, and endometriosis remain poorly understood, with limited treatment options. Many female-specific conditions, including menopause, endometriosis, and fibroids, are chronic conditions that fall under the purview of multiple ICOs and there are currently few funding opportunities targeting these conditions.

**Fig. 3:**
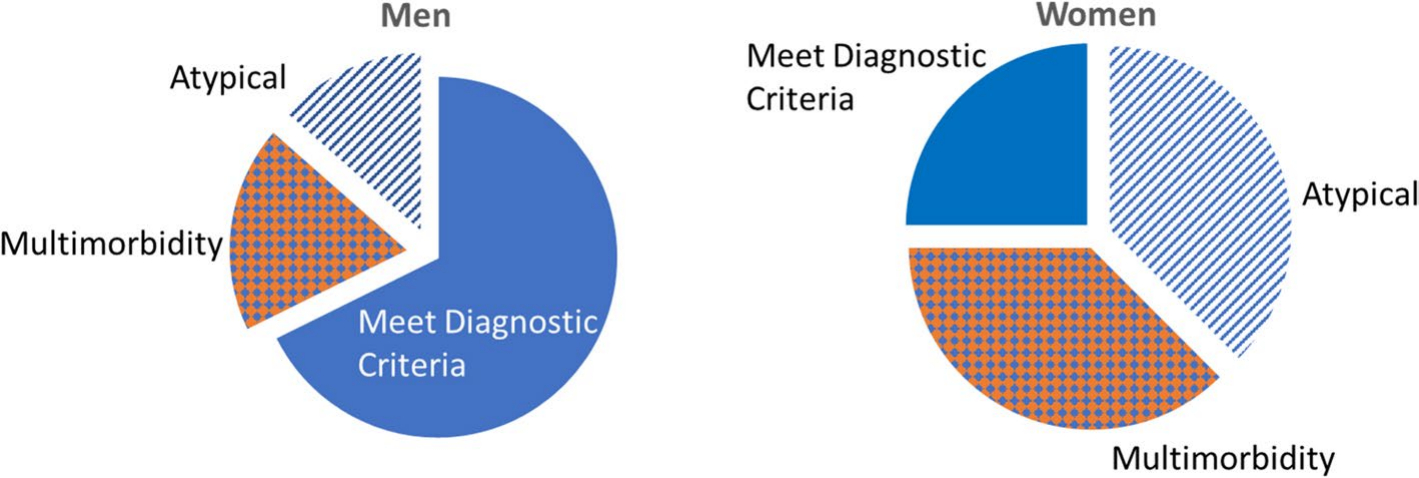
Exclusion of subjects with multimorbidities decreases the validity and generalizability of research throughout the pipeline. Disparate funding is a telescoping problem: The relevance of new data is focused on the population that we already know the most about; important disease-relevant, mechanistic information revealed by studying across populations is missed. Therefore less information is available on any given chronic condition in women, because fewer women meet the diagnostic criteria making them eligible for clinical research

Because women have higher rates of multimorbidity compared with men, challenges for research on multimorbidity have greater impact on the health of women. Research gaps include studies to better understand which subpopulations are affected disproportionately; population-level distributions of chronic diseases, particularly those specific to women; and studies that evaluate the interactions of multiple chronic diseases in women specifically. Without attention to multimorbidity, given that women are affected disproportionately by multiple chronic conditions, women then may be disproportionately excluded from clinical research because morbid conditions (e.g., chronic kidney disease) prevent women from meeting eligibility criteria (Fig. 3).

Studies that provide detailed sex-disaggregated and gender-specific clinical outcomes data—tied to critical life course windows, and hormonal transitions, from a diverse population of women, are needed to fill this critical research gap [67]. Large-scale prospective cohort studies of women might likewise begin to bridge gaps in understanding both the sex-specific pathophysiology and gender-specific influences on diagnosis, prevention, and treatment of chronic conditions in women. To fill these evidence gaps related to women’s health, clinical trials networks with the following could be established: a specific emphasis on women (including pregnant persons), tools to design trials that answer questions specific to women, expanded or extended eligibility criteria, and capacity – including dedicated funding support– to recruit and retain women of all ages and from diverse backgrounds in studies. Woman-focused clinical trials networks could partner with established NIH-supported clinical trials networks (e.g., NCI's National Clinical Trials Network (NCTN), NIAID’s HIV clinical trials networks, NIDA’s Clinical Trials Network (CTN)) to develop and support woman-specific trials, substudies, and retrospective analyses within these networks’ existing infrastructure. Finally, NIH could leverage longstanding prospective cohorts for existing, banked biospecimens and electronic medical or other virtually stored data to promote investigation of female-specific conditions and woman-centered research questions.

## Conclusion

Rising rates of chronic conditions represent a significant public health challenge, particularly relevant to the health of women. This challenge is exacerbated by the absence of a framework for characterizing and addressing chronic debilitating conditions in women. A review of NIH research activities on chronic conditions in women, completed in preparation for the congressionally directed “Advancing NIH Research on Women’s Health” conference, revealed multiple opportunities to align the NIH research agenda with the health needs of women. Social determinants of health, including factors such as gender, race, ethnicity, socioeconomic factors, and healthcare access, influence the health of women differently than the health of men; the role of social determinants of health on women’s burden of chronic conditions warrants further research and consideration. The 2016 SABV Policy has enhanced preclinical and discovery knowledge of relevant diseases; however, the impact of sex as a biological variable on chronic conditions in women across the life course, including during key hormonal transitions like menarche and menopause, is inadequately understood. Research that utilizes a life course approach, is informed by gender considerations, and incorporates systematic consideration of sex differences aligns with a global agenda for women’s health [68].

## Data Availability

The datasets generated and/or analyzed during the current study are available in the NIH and IHME repositories, https://reporter.nih.gov/ and https://vizhub.healthdata.org/gbd-results/.

## Abbreviations

ACRWH: Advisory committee on research on women’s health
CMS: Centers for medicare and medicaid services
COPD: Chronic obstructive pulmonary disease
CTN: Clinical trials network
CVD: Cardiovascular disease
DALYs: Disability-adjusted life years
FY: Fiscal year
HHS: Department of health and human services
ICO: Institute, center or office
MHT: Menopausal hormone therapy
NCI: National cancer institute
NCTN: National clinical trials network
NHANES: National health and nutrition examination survey
NHIS: National health interview survey
NHLBI: National heart, lung, and blood institute
NIA: National institute of aging
NICHD: *Eunice Kennedy Shriver* national institute of child health and human development
NIGMS: National institute of general medical sciences
NIH: National institutes of health
NIMHD: National institute on minority health and health disparities
OBSSR: Office of behavioral and social sciences research, and the office of disease prevention
OD: Office of the director
ODP: Office of disease prevention
ORHW: Office of research on women’s health
PCOS: Polycystic ovarian syndrome
RCDC: Research, condition, and disease categorization
SABV: Sex as a biological variable
SCORE: The specialized centers of research excellence
WHI: Women's health initiative
WHO: World health organization
